# Outcomes of Civilian Penetrating Rectal Injuries Associated With Genitourinary and Bony Injuries

**DOI:** 10.1002/wjs.70041

**Published:** 2025-08-13

**Authors:** Terron Govender, Zahra Abrahams, Deidre McPherson, Sorin Edu, Andrew Nicol, Pradeep Navsaria

**Affiliations:** ^1^ Trauma Centre Groote Schuur Hospital University of Cape Town Observatory Cape Town South Africa

**Keywords:** bladder, extraperitoneal, intraperitoneal, penetrating trauma, rectum

## Abstract

**Background:**

Combined rectal, bladder, and bone injuries are rare but associated with significant morbidity. This study aims to evaluate the outcomes of such injuries.

**Methods:**

A retrospective review of patient records was conducted for all individuals with full thickness penetrating rectal injuries admitted to the Trauma Centre at Groote Schuur Hospital between January 2010 and December 2019. Intraperitoneal rectal injuries were repaired, whereas extraperitoneal rectal injuries were left untreated, with a diverting loop colostomy performed. Intraperitoneal bladder injuries were repaired, whereas extraperitoneal bladder injuries were repaired through cystostomy. Pelvic and spinal fractures were irrigated, but presacral drainage and distal rectal washouts were not performed. Infectious complications and mortality were documented.

**Results:**

A total of 104 patients with 134 rectal injuries were identified (10 intraperitoneal, 64 extraperitoneal, and 30 combined injuries). Genitourinary tract injuries were identified in 42 (40.38%) patients, and 50 patients (48.08%) had associated bone injuries, including sacral (22), iliac (9), pubic rami (5), coccygeal (1), acetabular (3), femoral (6), and pelvic joint (5) fractures. A total of 91 diverting loop colostomies and three Hartmann's procedures were performed. Nine fistulae (6.7%) were observed, including three rectocutaneous, three rectovesical, one small bowel cutaneous, one vesicocutaneous, and one enteroenteric. There were 27 infectious complications, including 13 surgical site infections, 2 cases of pelvic osteitis, and 12 soft tissue infections.

**Conclusion:**

Extraperitoneal rectal injuries with associated bladder and/or bone injuries can be safely managed with fecal diversion, extraperitoneal bladder repair through cystostomy, and irrigation of bone and joint injuries, with minimal morbidity.

## Graphical abstract

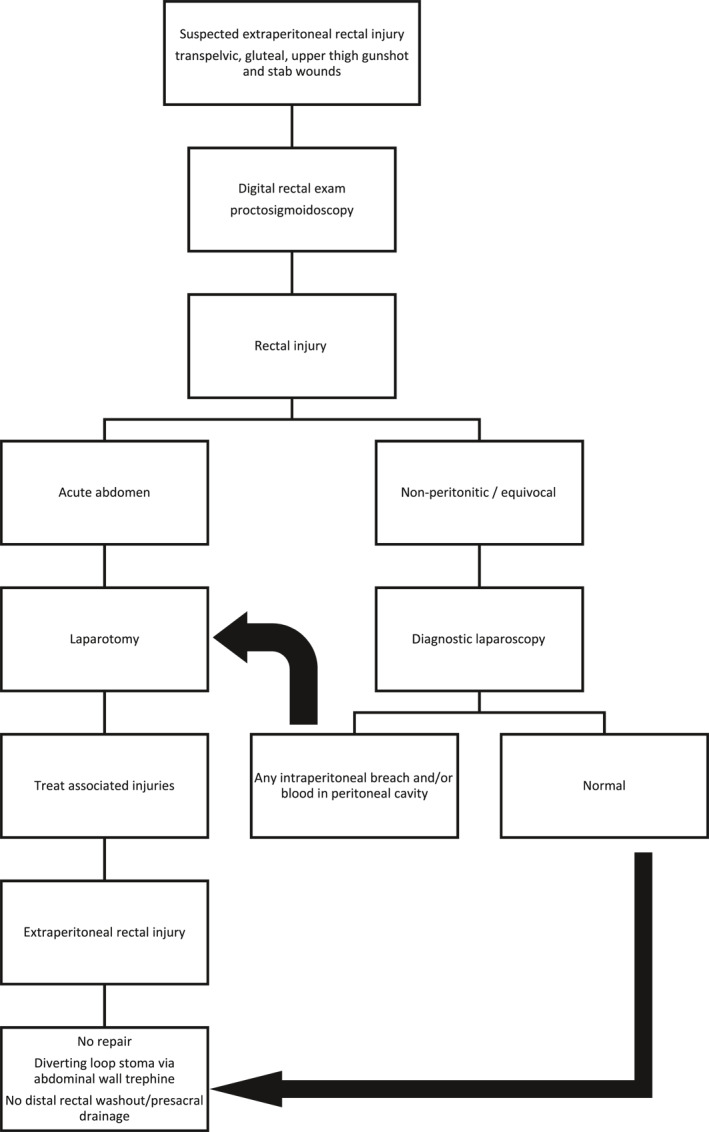

## 1 Introduction

The narrow pelvis, containing the rectum and adjacent vital structures, presents a unique challenge in trauma surgery, particularly when managing penetrating injuries. Historically, the military dogma of the “four D's”—*d*ebridement, *d*iversion, *d*istal washout, and *d*rainage—dominated management paradigms for penetrating rectal injuries. However, civilian trauma data have since challenged the necessity of all four components, demonstrating that extraperitoneal rectal injuries (EPRI) can be managed effectively with fecal diversion alone, avoiding the morbidity of distal rectal washouts and presacral drains [1, 2, 3, 4]. Despite this evolution, outcomes associated with concomitant genitourinary (GU) and orthopedic injuries, commonly seen in patients with transpelvic missile trajectories, remain underreported. These associated injuries may complicate management decisions and increase morbidity, yet no consensus exists on whether current algorithms adequately address them. This study aims to evaluate a decade of experience in managing penetrating rectal injuries at a high‐volume civilian trauma center. We focus specifically on outcomes in patients with associated GU and orthopedic injuries, in the context of a consistent institutional treatment algorithm.

## 2 Methods

### 2.1 Study Design and Setting

This was a retrospective cohort study conducted at a tertiary academic trauma center. Records of all patients with a penetrating rectal injury between January 1, 2010, and December 31, 2019, were reviewed. The institutional trauma database and operative notes were used for data extraction. Ethics approval was obtained from the institutional review board.

### 2.2 Inclusion and Exclusion Criteria

All patients with penetrating rectal trauma were eligible. Patients who died before definitive rectal injury management or underwent damage control laparotomy were excluded.

### 2.3 Data Collection

Demographic data, injury mechanism, and clinical parameters (vital signs, Revised Trauma Score [RTS], Injury Severity Score [ISS], Penetrating Abdominal Trauma Index [PATI], and Trauma and Injury Severity Score [TRISS]) were collected. The anatomical location of rectal injury—intraperitoneal (IPRI), extraperitoneal (EPRI), or combined—was determined intraoperatively. Imaging and preoperative assessments were considered, but intraoperative findings took precedence when discrepancies existed.

The trajectory of gunshot wounds (GSWs) was inferred from wound location, intraoperative findings, and the treating surgeon's assessment. Cross‐sectional imaging was used in hemodynamically stable patients. The presence of associated GU injuries (e.g., bladder trauma) and bone or joint injuries was recorded.

### 2.4 Management Protocol

Management adhered to Advanced Trauma Life Support (ATLS) principles and a published institutional algorithm [1]. All patients with missile trajectories near the pelvis underwent digital rectal examination (DRE) and rigid sigmoidoscopy (RS). Hemodynamically unstable patients or those with peritonism underwent exploratory laparotomy. IPRI were repaired primarily, with or without diversion. EPRI were diverted with loop sigmoid colostomy without repair or adjuncts. Selected patients underwent diagnostic laparoscopy prior to diversion through trephine.

Presacral drainage and distal rectal washouts were not used. Bladder injuries were managed according to location: Intraperitoneal bladder injuries (IPBI) underwent primary repair. Extraperitoneal bladder injuries (EPBI) were managed by transvesical repair through cystotomy. Bone and joint injuries were irrigated following abdominal exploration.

**FIGURE 1:**
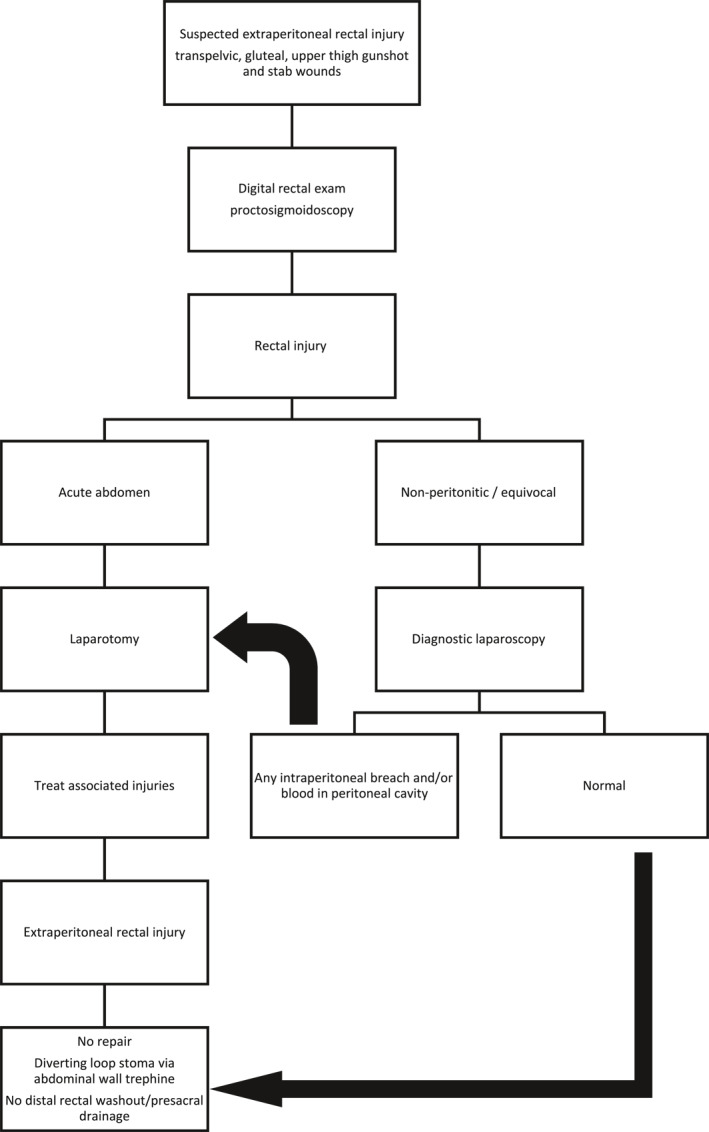
Management algorithm for low velocity penetrating extraperitoneal rectal injuries. Reproduced from Navsaria et al. [1].

Empiric antibiotic therapy was initiated in all patients. Antibiotic selection varied by attending physician, though most received amoxicillin–clavulanic acid (Figure 1).

### 2.5 Outcomes

Primary outcomes included postoperative complications (surgical site infection [SSI], intra‐abdominal sepsis, and fistula formation), length of hospital, and mortality. Secondary outcomes assessed the nature and management of associated injuries.

### 2.6 Data Analysis

Data were compiled in the spreadsheet software (Numbers for macOS). Continuous variables are presented as means with ranges and categorical data as frequencies and percentages.

## 3 Results

### 3.1 Patient Characteristics

Of 110 patients identified with rectal injuries, 6 were excluded due to damage control laparotomy, leaving 104 for analysis. The cohort was predominantly male (93.3%, *n* = 97) with a mean age of 28.4 years (range: 15–56). GSWs accounted for 101 injuries and stab wounds for three. Transpelvic trajectories (between iliac crests and perineum) were documented in 75 patients (72.1%). Twelve patients (11.5%) presented with hemorrhagic shock, all of whom responded to fluid and blood product resuscitation without requiring operative hemorrhage control.

### 3.2 Injury and Diagnostic Details

**FIGURE 2:**
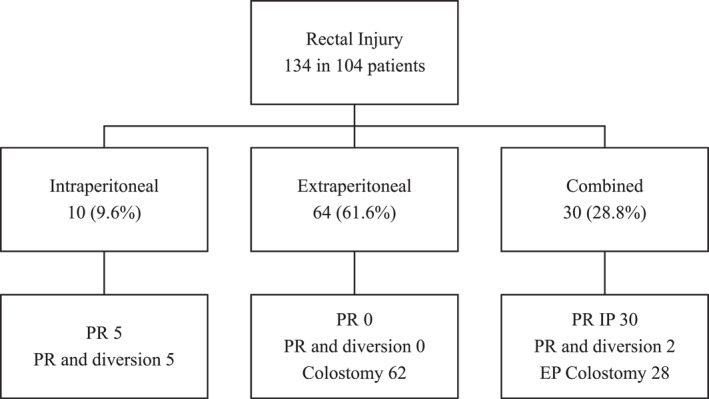
Overview of rectal injuries and their management.

Digital rectal examination was positive in 55 of 100 performed (55%). RS was performed in 100 patients; 96 (96.0%) showed evidence of injury. Hematuria was present in 65 patients (62.5%). Contemporary trauma scores are presented in Table 1. A total of 134 rectal injuries were documented in the 104 patients: 10 IPRI, 64 EPRI, and 30 combined injuries. Thirty IPRI were repaired primarily. Eighty‐nine EPRI were managed with loop sigmoid colostomy. No patient underwent presacral drainage or distal washout (Figure 2).

**TABLE 1:** Patient demographics, injury characteristics, and associated injuries (*N* = 104).

Sex	Female: 7 and male: 97 (93.27%)
Age	28.40 (IQR: 13) years
Mechanism of injury	Gunshots: 101 and stabs: 3
Transpelvic trajectory	75 (72.12%)
Shock on presentation	12 (11.54%)
Peritonism on presentation	65 (62.50%)
Trauma scores	
Injury severity score	19.02 (IQR: 5)
Penetrating abdominal index	19.59 (IQR: 11)
Revised trauma score	8.23 (IQR: 0)
Trauma and injury severity score	99.90 (IQR: 0.0385)
Small bowel	52 (50, 00)
Large bowel	17 (16, 35)
Genitourinary tract	42 (40, 38)
Kidney	3 (2, 88)
Ureter	7 (6, 73)
Bladder	27 (25, 96)
Intraperitoneal	9 (8, 65)
Extraperitoneal	4 (3, 85)
Combined	14 (13, 46)
Urethra	2 (1, 92)
Genitalia	3 (2, 88)
Bone and joint space	50 (48, 08)
Sacrum	22 (21, 15)
Coccyx	1 (0, 96)
Vertebra	3 (2, 88)
Iliac	9 (8, 65)
Pubic rami	5 (4, 81)
Acetabulum	3 (2, 88)
Femur	6 (5, 77)
Pelvic joints	5 (4, 81)
Vascular	8 (7, 69)
External iliac vein	2 (1, 92) (ligated)
Internal iliac vein	2 (1, 92) (ligated)
External iliac artery	2 (1, 92) (primary repair)
Internal iliac artery	2 (1, 92) (ligated)

### 3.3 Associated Injuries

**FIGURE 3:**
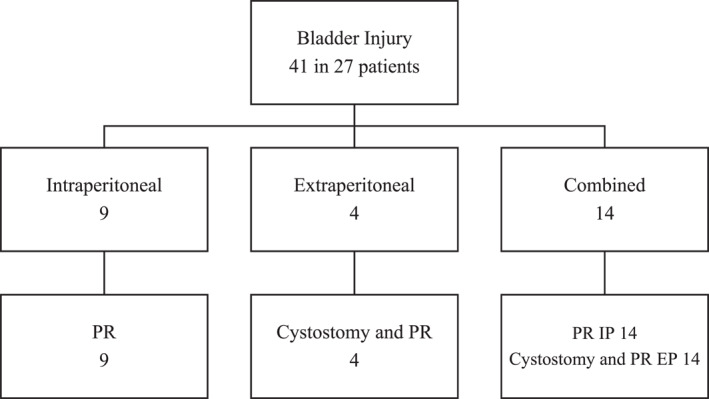
Overview of associated bladder injuries and their management.

**FIGURE 4:**
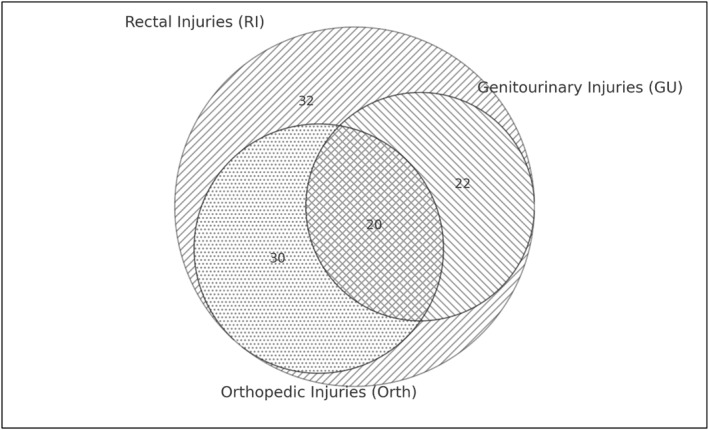
Venn diagram illustrating the distribution and overlap of rectal, genitourinary, and orthopedic injuries among 104 patients with full thickness penetrating rectal injuries. A total of 32 patients sustained isolated rectal injuries, 22 had combined rectal and genitourinary injuries, 30 had rectal and orthopedic injuries, and 20 had all three injury types. No patients presented with genitourinary or orthopedic injuries in the absence of rectal injury. Line patterns are used for shading to ensure clarity in print and grayscale formats.

GU injuries were present in 42 patients (40.4%), including 27 bladder injuries (26.0%). Of these, 9 were intraperitoneal, 4 extraperitoneal, and 14 combined. Figure 3 shows the management of the bladder injuries. Orthopedic injuries were identified in 50 patients (48.1%). A Venn diagram (Figure 4) illustrates overlap between rectal, GU, and orthopedic injuries (Table 1).

### 3.4 Antibiotic Regimens

Most patients (49.0%) received amoxicillin–clavulanic acid. Others received triple therapy (ampicillin, gentamicin, and metronidazole; 40.4%), clindamycin/ciprofloxacin (9.6%), or ceftriaxone/metronidazole (1%).

### 3.5 Outcomes

Complications included 13 SSIs (12.5%), of which six were superficial and four deep. Three patients developed intra‐abdominal abscesses. Twelve soft tissue infections were recorded. Nine patients developed fistulae: 3 rectocutaneous, 3 rectovesical, and 3 other types. No mortality was recorded among the included cohort. Complications are summarized in Table 2. Mean hospital stay was 12, 3 (R3–79) days in patients with complications and 8.5 (R3–15) days without complications.

**TABLE 2:** Procedures performed for rectal injuries (*N* = 134) and complications.

Extraperitoneal rectal injuries (94)	
Loop sigmoid colostomy	84
Laparoscopic assisted loop sigmoid colostomy	4
Loop colostomy in different position	1
Primary repair and loop sigmoid colostomy	2
Hartmann's type procedure	3
Intraperitoneal rectal injuries (40)	
Primary repair	30
Primary repair and loop sigmoid colostomy	9
Hartmann's type procedure	1
Surgical site infection	13
Superficial	6
Deep	4
Intra‐abdominal	3
Soft tissue infections	12
Abscess	2
Necrotizing fasciitis	2
Presacral collection	3
Bullet tract infections	5
Fistulae	9
Rectocutaneous	3
Rectovesical	3
Vesicocutaneous	1
Enterocutaneous	1
Enteroenteric	1
Osteitis	2

## 4 Discussion

This study presents a 10‐year experience in managing civilian penetrating rectal injuries using a structured minimalist surgical approach. We found that EPRI can be effectively managed with fecal diversion alone and IPRI with primary repair in stable patients. Importantly, the omission of presacral drainage and distal washout did not result in increased infectious complications. Furthermore, combined bladder‐rectal trauma can be safely managed without separating suture lines and bone/joint injuries involving the rectum can be safely managed with thorough washout.

Rectal injuries (RIs) are rare and challenging to manage due to the complex anatomical relationships between the rectum and surrounding structures. The peritoneal reflections surrounding the rectum are incomplete, with the proximal third covered anteriorly and laterally, the middle third anteriorly, and the distal third extraperitoneal. This anatomical distinction is crucial in determining the appropriate surgical approach. Additionally, the rectum lacks a mesentery and is enveloped by the visceral pelvic fascia [5].

A review of rectal injuries in the United States reported an incidence of 0.1% [6]. In other series, 104 patients were documented over two years [7] and 92 patients over four years [1]. Penetrating trauma, particularly from low‐velocity firearms, remains the leading cause of rectal injury in the civilian setting [6, 8, 9]. Locally, a recent 2020 review found that 80% of rectal injuries were due to penetrating mechanisms (71% from firearms), with 14% resulting from blunt trauma and 6% from both penetrating and blunt mechanisms [2].

Traditionally, rectal injuries have been classified based on the mechanism of injury and the anatomical location. Extraperitoneal rectal injuries (EPRI) do not communicate with the peritoneal cavity, whereas intraperitoneal rectal injuries (IPRI) do. Combined injuries (CI) involve both intraperitoneal and extraperitoneal injury. Given the low incidence of these injuries and the lack of definitive clinical signs, diagnosing EPRI can be particularly difficult, with delayed diagnosis resulting in significant morbidity. Although DRE and rigid sigmoidoscopy (RS) are useful in detecting nonbenign lesions of the rectum, their sensitivity for identifying rectal injuries is suboptimal—51% and 78%, respectively [10]. This highlights the importance of early identification, particularly in patients with a transpelvic trajectory in penetrating trauma. For those without indications for an expedited laparotomy, cross‐sectional imaging should be performed to assess injury extent and associated damage. Standardized algorithmic management is crucial for optimizing patient outcomes.

Contemporary management of rectal injuries has evolved, with IPRI typically managed by primary repair and EPRI managed with diversion. Although presacral drainage (PSD) and distal rectal washout (DRW) have been proposed as treatment adjuncts, the true benefit of these interventions has not been conclusively demonstrated. Several studies have shown similar outcomes with or without their use [11, 12, 13, 14, 15, 16, 17]. In our practice, we have found the algorithm proposed by Navsaria et al. [1] to be effective and straightforward for managing these injuries (Figure 3). We believe routine DRW and PSD have no role in the modern management of rectal injuries. Drainage should be reserved for delayed presentations or patients with established infectious complications. Furthermore, DRW and PSD have been shown to be independent risk factors for developing intra‐abdominal sepsis [8].

Given the proximity of the urogenital tract, pelvic organs, and rectum, associated injuries in these regions should be carefully considered. Our study revealed a high frequency of associated genitourinary tract injuries (40.38%) and bone/joint injuries (48.08%). Earlier literature has reported similar rates, with one study documenting associated genitourinary injuries in 41.3% of patients [18] and another large multicenter review reporting 42% [19]. Our approach to managing bladder injuries, as detailed in the methods section, has been effective. We recommend that IPBI be managed with primary repair and EPBI injuries through cystostomy and repair from within the bladder, without the need for suture line separation between the repaired bladder and rectal injury, resulting in minimal sequelae.

Bone and joint injuries are also frequently associated with penetrating rectal injuries. In our series, 48.08% of patients had an associated bone injury. These injuries were thoroughly irrigated after managing the rectal and bladder injuries in the operating room, with only two patients developing osteitis. This suggests that our management approach is appropriate, although identifying missile trajectories that pass through the rectum first may help predict which patients are at higher risk for bone complications. Empiric antibiotic therapy with amoxicillin and clavulanic acid is appropriate as shown in our study. The small number of patients who received clindamycin and ciprofloxacin were treated during a period when empirical therapy for GSWs and bone fractures was based on the hospital's antibiotic protocol.

Though the risk of infection remains high in these patients, the majority sustained concurrent injuries to other organs and presented with overt peritonism. Therefore, complications should be viewed primarily in terms of surgical site infections and fistulae. The development of necrotizing fasciitis at the time of presentation is treated with a combination of antibiotics and surgery. The primary treatment is surgery, which involves removing all necrotic tissue. This may require multiple surgeries and extensive debridement of the infected area. Along with surgery, broad‐spectrum intravenous antibiotics are administered to target the bacteria. Retained missiles in the affected area are sought and removed, whereas the rectal and associated injuries are managed as usual.

Eight iliac vessel injuries were identified in the current cohort. Intra‐abdominal injuries vascular injuries with or without rectal trauma is managed the same. In the pelvis, injured external or internal iliac veins are either ligated or repaired if amenable to simple venorrhaphy. The internal iliac artery and accessible branches are ligated. The external iliac artery is repaired either primarily with end‐to‐end anastomosis or a reversed saphenous vein or prosthetic graft. The repair is always protected and covered with peritoneum, particularly in the presence of bowel contamination [20].

Nerve injuries, in particular obturator and sciatic nerve involvement is inferred based on meticulous clinical assessment. These are referred to the orthopedic department for long‐term follow‐up and management. Early physiotherapy and occupational therapy is initiated and continued as outpatient treatment. Similarly, vaginal and uterine injuries are promptly referred to the Gynecology Department. Suspected vaginal injures undergo EUA and suture through speculum. Uterine injuries are usually amenable to primary repair.

## 5 Limitations

This retrospective study is limited by potential documentation and selection biases. Variability in the application of rigid sigmoidoscopy and classification of injury level was influenced by trainee involvement. Antibiotic regimens and operative strategies reflected individual surgeon preferences within the institutional algorithm. Lastly, trajectory classification was subjective in the absence of ballistic studies.

## 6 Conclusion

Our data support a focused standardized approach to penetrating rectal injuries: EPRI can be managed by fecal diversion alone. IPRI can be repaired primarily in stable patients. Combined bladder‐rectal trauma can be safely managed without separating suture lines. Bone/joint injuries involving the rectum require thorough washout. Adjuncts, such as presacral drainage and distal rectal washout, appear unnecessary and empiric use of amoxicillin–clavulanic acid is effective.

## Author Contributions

**Terron Govender:** data curation (equal), formal analysis (equal), writing – original draft preparation (lead). **Zahra Abrahams:** data curation (equal), formal analysis (equal). **Andrew Nicol:** writing – review and editing (equal), conceptualisation (support). **Sorin Edu:** writing – review and editing (equal). **Deidre McPherson:** writing – review and editing (equal), supervision (support). **Pradeep Navsaria:** conceptualization (lead), methodology (lead), project administration (lead), supervision (lead), writing – review and editing (lead).

## Ethics Statement

This study was approved by University of Cape Town ethics: HREC 467/2018.

## Conflicts of Interest

The authors declare no conflicts of interest.
